# Risk Assessment and Community Participation Model for Environmental Asthma Management in an Elementary Public School: A Case Study in Puerto Rico

**DOI:** 10.3390/ijerph2006030009

**Published:** 2006-03-31

**Authors:** Samarys Seguinot-Medina, Alberto Rivera-Rentas

**Affiliations:** 1 Universidad Metropolitana, San Juan, Puerto Rico;; 2 Universidad del Turabo, Gurabo, Puerto Rico.

**Keywords:** Asthma, Environment, Prevalence, Schools, Community, Puerto Rico

## Abstract

Asthma is a rapidly growing chronic disease in the general population of the world, mostly in children. Puerto Ricans have the highest prevalence of children with asthma among the Hispanic community in the US and its territories. Asthma and air quality are becoming a significant and potentially costly public health issue in Puerto Rico. The CDC has reported that in Puerto Rico, 320,350 adults have asthma and this number represents 11.5% of the island adult population. The north east municipality of Carolina, Puerto Rico, has the highest asthma prevalence in the 0 to 17 year old range (2001 data). In this study, we address the potential relationship between anthropogenic and naturally occurring environmental factors, and asthma prevalence in an urban elementary public school in Carolina in an effort to empower and engage communities to work on their environmental health issues. We integrated geographic information systems (GIS) data of anthropogenic activities near the school as well as the natural resources and geomorphology of the region. We found that as Carolina is close by to Caribbean National Forest (El Yunque), this together with the temperature and precipitation cycles in the zone creates the ideal environmental conditions for increased humidity and pollen, mold and fungi development through out the year. We also collected health and socio economic data to generate an asthma profile of the students, employees and parents from the school community, and through a survey we identified perceptions on environmental asthma triggers, and indoor air quality in the school and homes of the students and employees. Finally, we implemented a workshop on indoor air quality designed to engage the school community in managing asthma triggers and the school environment. Our results showed that nearly 30 % of its student’s population has asthma, and from this group 58% are males and 42% are female students. Of all asthmatic children, only 43% receive treatment for the disease. The study also showed that most asthmatic children are between 7 and 9 year old, and live in households with an annual income below $10,000. It also showed that 25 % of the student’s parents have the condition, and that 25% of the employees are also affected by this chronic condition. All these numbers are significantly higher than those reported by the CDC for Puerto Rico. The perception component had a response of 83% of school employees, and a 39% response from parents. It showed that people know asthma as a disease but many can’t identify most environmental asthma triggers. Pre and post tests of the workshop protocol showed that before the activity only 21% of participants can identify asthma triggers. At the end of the workshop nearly 80% were able to identify and manage environmental asthma triggers. This work validates the fact that Puerto Rico continues to have a significant number of people with asthma, particularly children asthma, and that schools are an important settings to create community based action plans to manage environmental asthma triggers through outreach and training.

## Introduction

Asthma is one of the most common chronic diseases in the world, with reported estimates as high as 300 million people having this condition. This chronic disease is responsible for one (1) per every 250 deaths worldwide and it is estimated that by 2025 over 100 million persons will have asthma [1]. This respiratory condition is the most common chronic disease amongst children. In the United States it affects over 8.6 million children and it has the highest prevalence among children between five (5) and 17 years old [2]. In addition, the Center for Disease Control (CDC) reported that in the US asthma related deaths of children 19 or younger increased to 78% between 1980 and 1993 [3]. The costs of medical services related to this condition have been increasing as well, and by 1994 the estimated total costs overcome 9.8 billions in the US [4].

The CDC data show that minority underrepresented groups tend to show a higher asthma prevalence than white groups. The adult asthma prevalence in the US is 7.5% and the prevalence per ethnic groups is as follows, whites (7.6), African American (9.05), and Hispanics (7.7). CDC data showed that, of all US states and territories, Puerto Rico has the highest overall prevalence of lifetime (19.6%) and current (11.6%) asthma. This difference is also shown in the asthma prevalence in children, as in the US it is reported to be 7.2% and in Puerto Rico is reported to be 33.2%. Beckett [5] reported that amongst families that have at least one child with asthma, 24.5% were mainly Puerto Rican, 17.7% African-american, and 9.6% white. Carter-Porkas and Gergen [6] reported that of the estimated half a million Hispanic children with asthma in the US, 75% of them are Puerto Ricans. Lara [7] reported that Puerto Rican children living in the US showed higher asthma prevalence than Mexican-american and Cuban-american children. They also have lower economic income and higher number of single mother households. Other studies reported that regardless the ethnic origin, Puerto Rican children living in the US show a higher prevalence of asthma and live in household environments with potential asthma triggers such as rats, roaches, dust and air and heat systems without maintenance [8]. Ledogar [9] measured asthma prevalence among Latinos of different cultural traditions who live on the same streets and in the same buildings in New York City (NYC) and reported that asthma period prevalence was 5.3% among Dominicans and other Latinos, compared with 13.2% among Puerto Ricans, a difference not explained by location, household size, use of home remedies, educational attainment, or country where education was completed.

Research data suggest that Puerto Ricans are more affected by the condition than other groups. Interestingly, almost all research studies have being performed with Puerto Ricans living in the US. Only a few studies have been completed in the island. The 2000 Behavioral Risk Factor Surveillance System (BRFSS) showed that Puerto Rico had the highest self-reported prevalence of asthma [10]. In the later report, the prevalence of asthma in any children interviewed was 33.2%, and only 51.3% were receiving treatment. Nazario [11] completed a parent response survey study in schools in the metropolitan area of San Juan and reported that significantly more children in public schools than in private schools have asthma (32.6% vs. 23.7%). Roure [12] completed an indoor air quality study in rural homes and reported increased asthma prevalence in homes with dust, pet dander, and humidity. Other indoor air quality studies include Montealegre [13] that studied children exposure to indoor endotoxins and allergens, and Milan [14] that studied house dust mites and asthma. Population-based studies in hundreds of cities in the U.S. and around the world have demonstrated a strong link between elevated particulate levels and premature deaths, hospital admissions, emergency room visits, and asthma attacks [15]. Groundbreaking long-term studies of children’s health conducted in California have demonstrated that particle pollution may significantly reduce lung function growth in children [16]. Gent and other scientists [17] found that ozone levels, but not particulate matter (PM) was significantly associated with respiratory symptoms and rescue medication use among children using maintenance medication.

It is known that asthma has both genetic and environmental causes and that this condition is one of the best examples of gene-environment interactions. Studies completed to understand genetic mechanisms causing asthma include studies in NYC by Colp [18] and in Connecticut by Crain [19] that suggest a potential relationship between high asthma prevalence among Puerto Rican children and variants of alpha1 anti-trypsin. In addition, the mechanisms underlying environmental asthma remains to be clarified. Asthma triggers have been identified for indoor and outdoor settings as well as the potential relationship between both settings and their air quality characteristics [20]. This is significant considering the increase of children with asthma worldwide, the reported asthma prevalence in Puerto Rican children, and the relationship between indoor and outdoor air quality. The studies completed in Puerto Rico contribute to the treatment of the condition and prevention of indoor pollutants. However, more work is needed to prevent children asthma and research in outdoor air quality will be crucial to understand environmental asthma.

The Department of Health of Puerto Rico has an ongoing project entitled Asthma Continuum Project that reports amongst other conditions, asthma prevalence for the 78 municipalities in the island. In 2001, the project reported that the municipalities in the central eastern region of Puerto Rico amongst the top 20 in asthma prevalence in the age range of 0 to 17. The north east municipality of Carolina, Puerto Rico, has the highest asthma prevalence in the 0 to 17 year old range. In this study, we will address the potential relationship between anthropogenic and naturally occurring environmental factors, and asthma prevalence in an elementary public school in Carolina in an effort to empower and engage communities to work on their environmental health issues.

## Methodology

This work is the first study completed in a public school setting in Puerto Rico to examine the potential relationship between environmental factors, asthma and community perceptions in an effort to develop a community based initiative to manage the school’s environmental health issues. In order to complete this study we created five data profiles:

(1) An environmental profile of the school including the natural resources surrounding the area, a geographic description of the region, anthropogenic activities close to the school’s location, and climate and weather data for the region;
(2) An infrastructure profile of the school describing the physical facilities, and their maintenance;
(3) A student profile including socioeconomic and health data such as gender, age, location, household financial income, home locations, and asthma diagnosis based on the school student record;
(4) A school’s teachers and administrative staff profile including socioeconomic data similar for those of the students except that health data was self reported;
(5) A students’ parents profile similar to that described in (4).

Environmental, climate and anthropogenic activities in the school’s area were analyzed using aerial photos, records from the National Weather Office in San Juan and NOAA, and geographic information systems (GIS) using ArcView and ArcGIS software. A site visit with a certified Registered Environmental Manager (REM) completed the school physical infrastructure profile. The students profile was completed for 643 students that is the total of the school student enrollment. Students’ socioeconomic and health data was collected from each student profile in the school after certified approval of the school’s administration and complying with HIPAA guidelines. Thus, no names and/or specific addresses were collected for this study. For location purposes only the home location in the community (better known as “barrio”) was recorded. A 40 items survey was designed to examine school’s teachers and administrative staff as well as parents’ perception of their knowledge of asthma, environmental asthma trigger s, and environmental asthma management and prevention at home and in the classroom. The latter was accomplished by implementing an indoor air quality tools for schools (IAQTFS) workshop in the school for teachers and administrative staff. The workshop facilitated the distribution and participation of administrative staff in the pre and post test as well as in the survey component of this study. Parent’s perception survey was distributed through the students that returned it to the teachers after the parents completed it. A community participation plan for asthma management was developed based on the results of this survey.

## Results and Discussion

**Figure 1:**
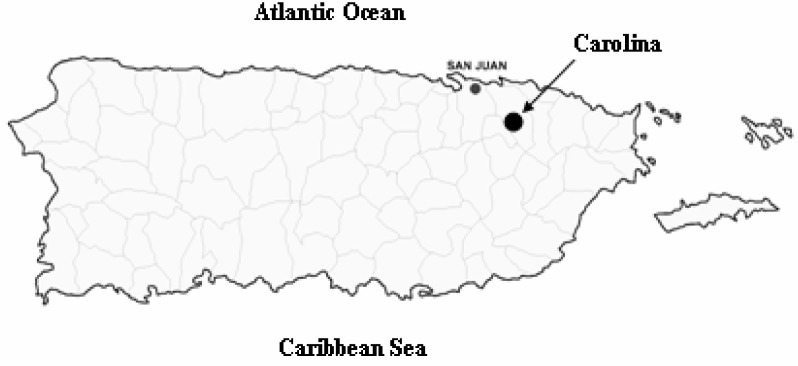
Map illustrating the geographic location of the school that participated in this study. School was located in Carolina, Puerto Rico, a municipality that has the Atlantic Ocean on the north, the Caribbean National Forest on the east, the Sierra de Luquillo mountains on the South and the capital of Puerto Rico, San Juan, on the west.

**Figure 2:**
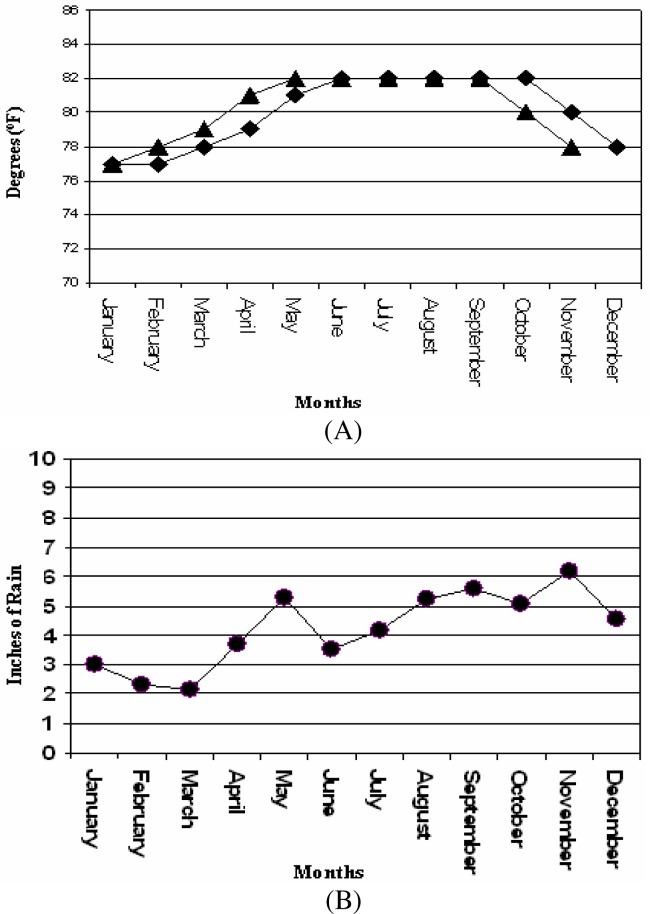
Temperature (A) and precipitation (B) profile for the municipality of Carolina, Puerto Rico. Data was kindly provided by the National Weather Service and the National Oceanic and Atmospheric Agency (NOAA) through the website.

This work is the first study of environmental asthma triggers completed at a school setting in Puerto Rico. The only study completed in Puerto Rico that is related to this work was reported by Nazario [21] that addressed parents reported child asthma and school setting. This report did not examine environmental triggers of asthma. The elementary public school that participated in this study is located in an urban –suburban setting in the municipality of Carolina, Puerto Rico. Carolina is located in the northeastern region in the island close to El Yunque National Reserve Rain Forest that is located in the east of the municipality (Figure 1). El Yunque is the largest rainforest in Puerto Rico. Temperature data (Figure 2A) gathered from the National Weather Service (NOAA) showed that Carolina has an almost constant temperature range between 76 and 82 degrees Fahrenheit.

Precipitation data for the municipality provided by the National Weather Service (NOAA) showed that Carolina gets between 2 to 6 inches of rain thorough out the year (Figure 2B). The precipitation profile of Carolina showed it has a dry season between January and April, and a wet season between May to December. The predominant wind pattern for Puerto Rico is eastward. The urban – suburban school location is characterized by several human activities that can be potentially related to asthma and or being contributing factors to asthma triggers.

**Figure 3:**
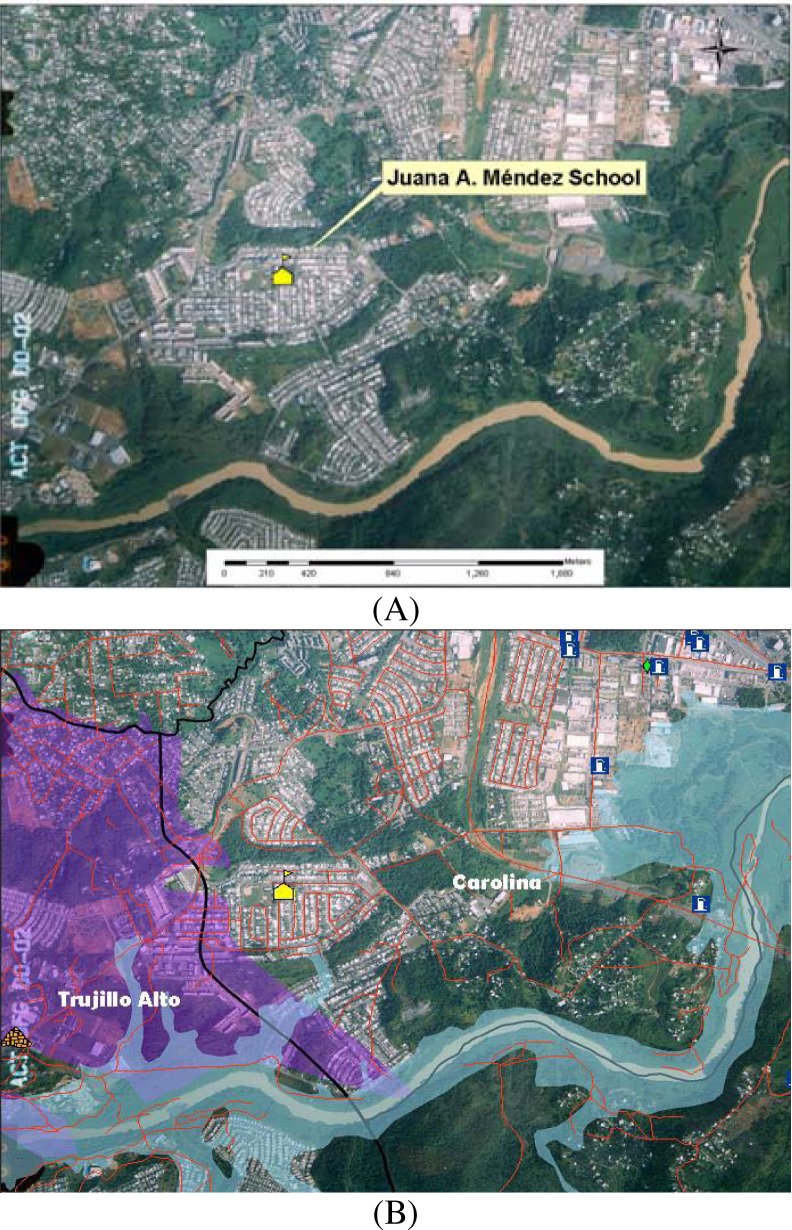
Aerial photos and maps illustrating the geographical and spatial relationship between the school setting and the location of anthropogenic and natural resources in the region in A. In B, the main industrial area is highlighted (in purple).

Figure 3A shows that the school is enclosed within a middle class housing community that includes several secondary streets. This school has additional communities and small forest areas on the north, and the east sides. Figure 3B shows that farther to the east there are several fuel stations. On the south side there are additional communities, some forest areas and the Rio Grande de Loiza River. Figure 3B also illustrates that on the west side the school is close to the largest industrial area in Carolina.

The school was built in 1990 and structurally includes a two (2) story concrete building. The building did not have any wall cracks or water leakages on the ceiling or the plumbing. It has 30 rooms including classrooms, computer room, arts and craft room, cafeteria, library, and administrative offices. Most of the classrooms do not have window screens to prevent pest entrance and since there are no air conditioning system almost all classrooms has floor, wall or table fans. The school has 12 bathrooms that are clean up during working hours and during our visits we observed that the floors are usually wet. There are only four air conditioning (AC) units in the school, three are functional and located in the kinder, pre kinder and computer rooms while the fourth was at the library. The latter was not functional or repaired while this study was completed (six months). The AC maintenance service is done by a contractor but in the duration of this study no maintenance was observed. The school has an interior yard cover almost completely (nearly 97%) with concrete and it has two trees. It also has a 50 car concrete parking lot surrounded by grass that is mown during school working hours. A team of U.S. and European scientists recently found that mowed grass emits hydrocarbons at a level of 20 to 60 parts per billion, which is comparable to the level released by the gasoline-powered mowers cutting the grass [22]. This team reported that common lawn mowing releases substantial amounts of reactive volatile organic compounds (VOCs) and should be considered in urban air-quality control strategies.

In this study we integrated geographic information systems (GIS) data of anthropogenic activities near the school as well as the natural resources and geomorphology of the region, with demographic and health data from the school community. We collected health and socio economic data to generate an asthma profile of the students, administrative staff and parents from the school community, and through a survey we identified perceptions of the environmental asthma triggers, and indoor air quality in the school and homes of the students and employees. Finally, we implemented a workshop on indoor air quality designed to engage the school community in managing asthma triggers and the school environment. We evaluated a total of 643 student data profiles to generate the school student population socio demographic and health profile. A total of 54 school employees (including teachers and administrative staff) received the survey and 45 (83.3%) participated and completed the survey study. A total of 592 surveys were distributed to the parents through the students, 230 parents returned a completed survey for a 39% total response.

**Figure 4:**
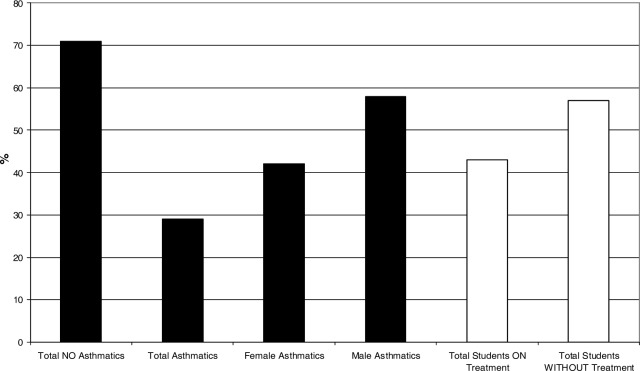
Profile of asthmatic students per gender and in treatment.

**Figure 5:**
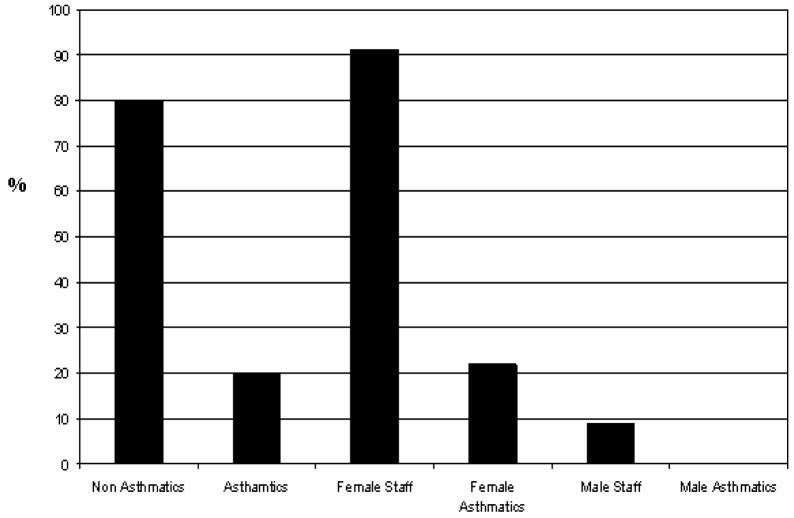
Profile of school’s administrative personnel (including teachers) with asthma per gender.

Our results in figure 4 show that nearly 30 % of the student’s population has asthma, and from this group 58% are males and 42% are female students. This revealed that male students are more affected by this condition than female students. This contrast significantly with parents and school administrative personnel results that showed that females are more affected by asthma than males. This gender and age difference should be further examined in terms of level of outdoor activity, genetic basis and treatment response. Of all asthmatic children, only 43% receive treatment for this chronic condition as illustrated in figure 4. The study also showed that most asthmatic children are between 7 and 9 years old, and live in households with an annual income below $10,000. The latter classifies the students’ families as under the poverty level using the United States bur eau of census. The profile of school administrative staff showed that the age range for the majority of employees is between 35 to 54 years old, have predominantly an academic background at the bachelor’s degree level, and their annual household income ranges $ 25,000 to $34,999. Figure 5 shows that 20% of the school staff is asthmatic and that more females than males are affected by this condition. The parents profile developed in this work indicates that most parents are within a age range between 25 to 34 years old, have an educational background at the high school level, and their annual household income is below $25,000.

**Figure 6:**
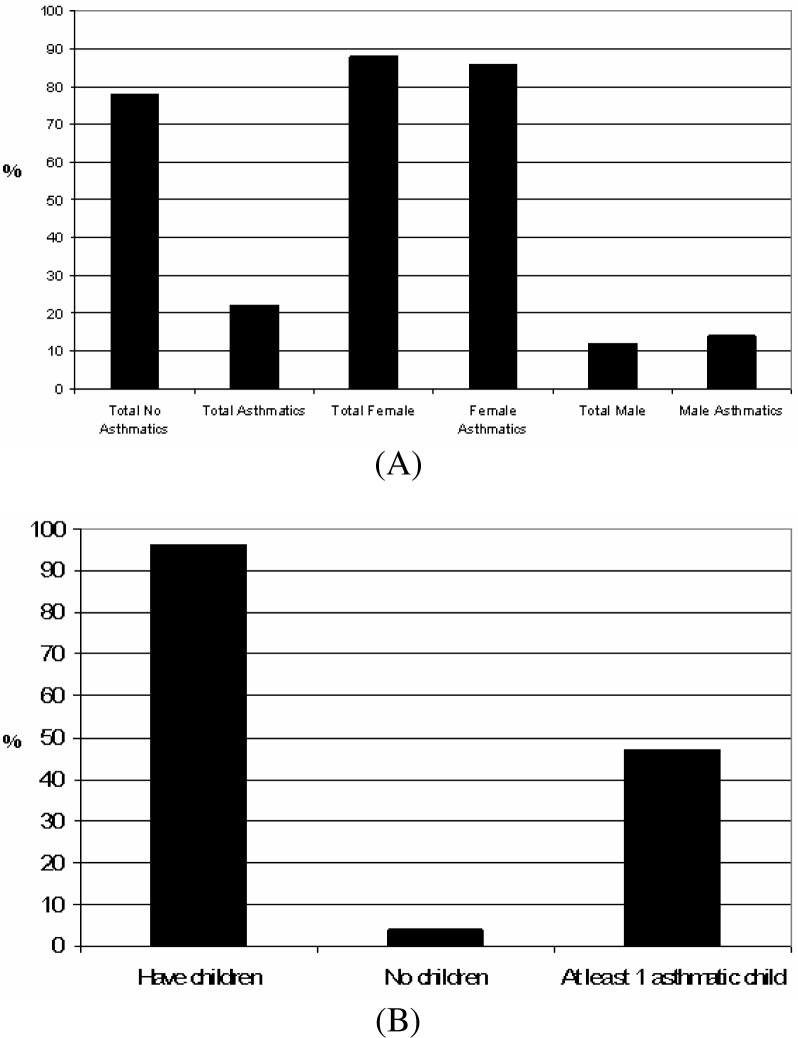
Profile of parents that self reported as asthmatic per gender (A) and those that have at least one (1) child with asthma (B).

However, the later fact can not be validated because over 25% of the parents did not report this data. But our data did reveal that parents and house heads of the students have lower academic backgrounds. Results shown in figure 6A indicate that 22% of parents are asthmatics and that similar to the employee profile, female parents are more affected by the condition (84%) than male parents (14%). In addition, results shown in figure 6B indicate that, regardless their asthma diagnosis, 47% of the participating parents have at least one (1) child with asthma.

**Figure 7:**
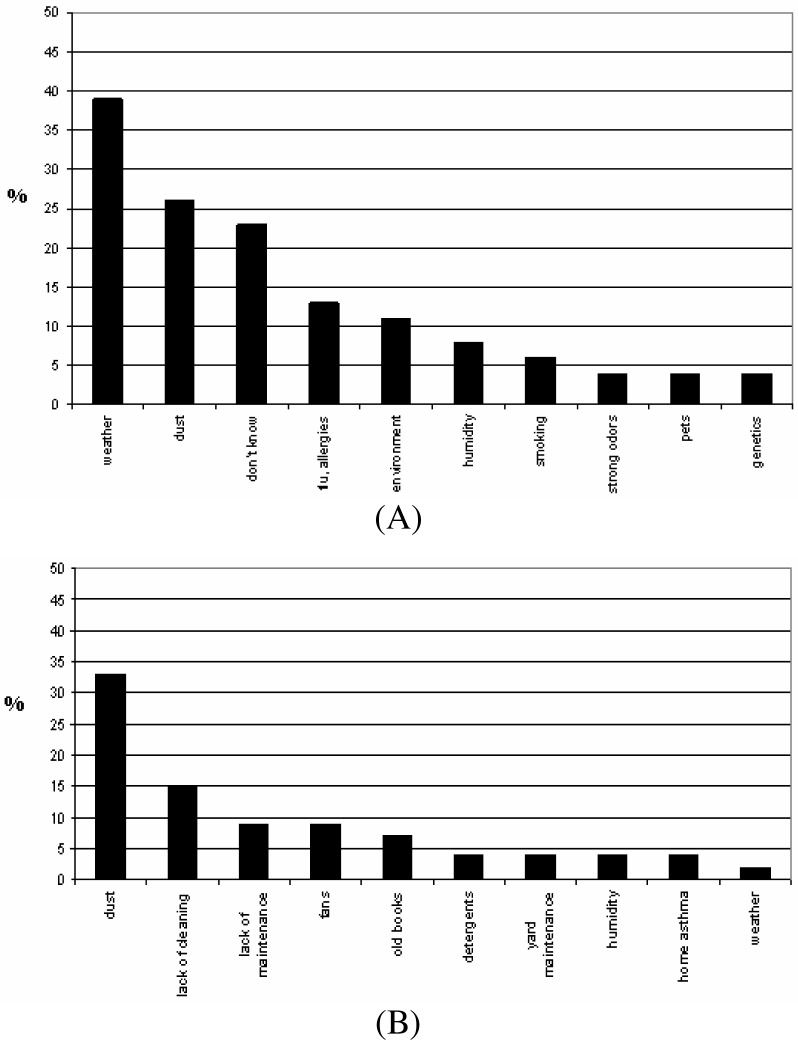
Profile of school’s administrative staff (including teachers) perceptions of asthma triggers in general (A) and in the classroom (B).

The perception component of this work, shown in figure 7A, revealed that surveyed parents identified weather and dust as the main causes of asthma attacks in their children, 39% and 26%, respectively. Interestingly, parents seem to separate weather and environment as the latter was identified by only 11%. School employees identified dust, strong odors and weather as the principal causes of asthma. Figure 7B illustrate that at the classroom level they overwhelmingly (over 30%) identified dust as the principal agent triggering asthma, followed by lack of cleaning and maintenance (15%, and less than 10%, respectively). Interestingly, humidity and weather were amongst the top ten causes but agents related to school infrastructure and maintenance represents potential strategies to address while developing an asthma management plan for the school.

**Figure 8:**
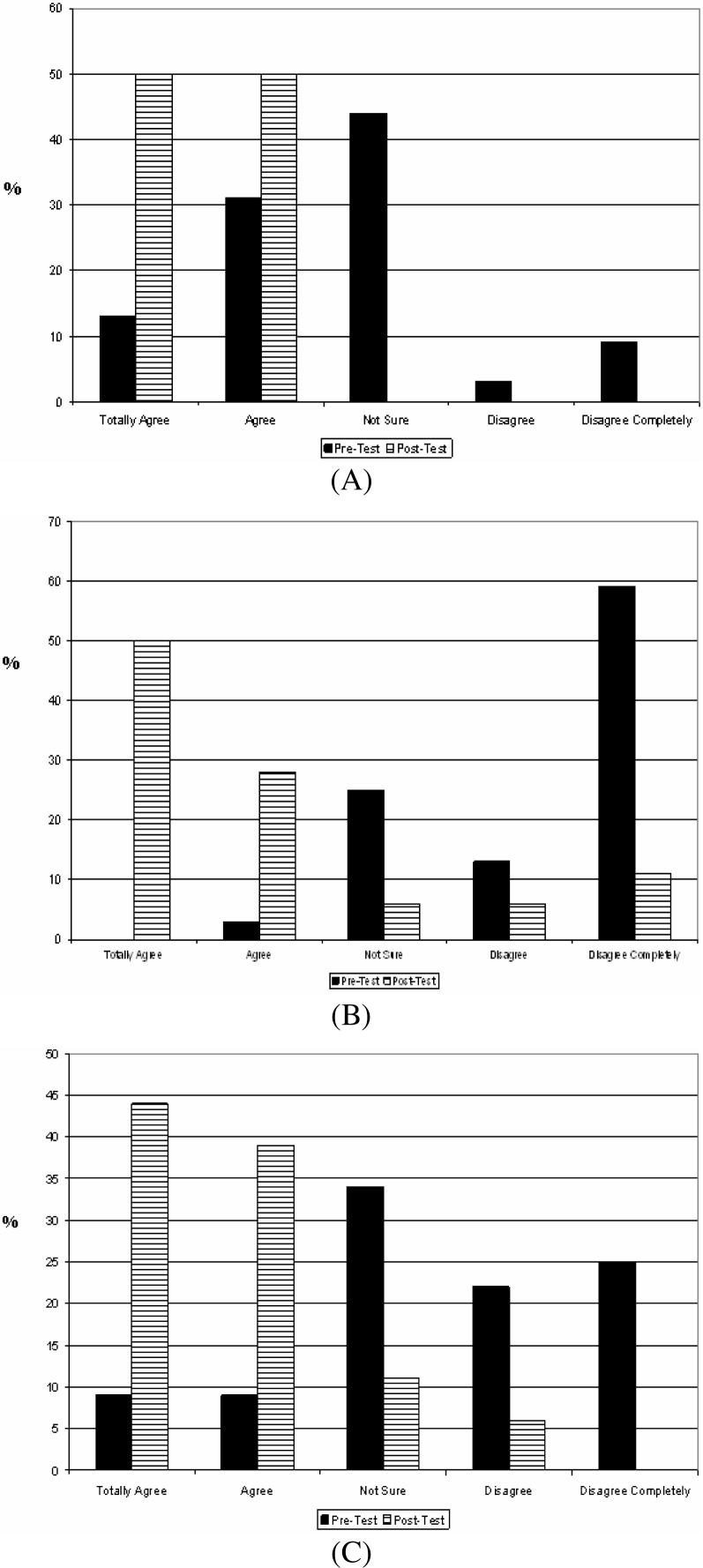

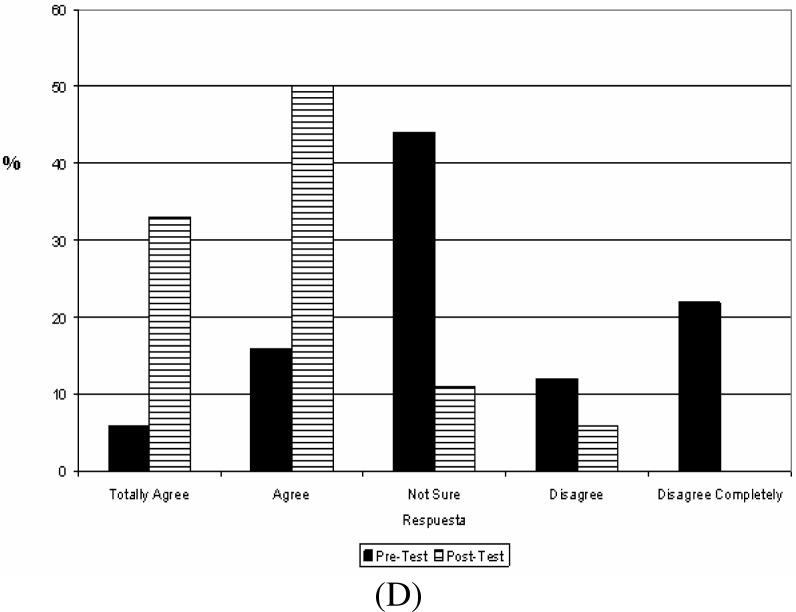
Results of the indoor air quality tool for schools (IAQTFS) training workshop pre and post tests. School’s administrative personnel (including teachers) responses to premises I know the method to control asthma triggers (A), I know what to do to avoid asthma attacks in the classroom (B); I know the impact of asthma on the students health and academic performance (C); and I know where to get information about indoor air quality (D).

The IAQTFS workshop offered to the school administrative staff included pre and post tests that demonstrated a significant impact on the level of knowledge on asthma and prevention strategies at the classroom level. Results of this work include:

(1) A 40% increase in the number of employees that perceived that after the workshop they have a better understanding of asthma;
(2) A 45% increase in the number of participants that agree that they better understand asthma triggers;
(3) 75% increased in the number of employees that perceived they better understand the methods to control asthma triggers (Figure 8A);
(4) 75% increase in the number of participants that have a better understanding on what to do to avoid asthma attacks in the classroom (figure 8B);
(5) A 75% increase in the number of employees that gained a better understanding of the impact of asthma on students academic performance and quality of life (Figure 8C); and
(6) A 61% increase in the number of participants that know where to find information about indoor air quality (Figure 8D).

Studies from United State and Europe show that persons in industrialized nations spend more than 90 percent of their time indoors [23]. For infants, the elderly, persons with chronic diseases, and most urban residents of any age, the proportion is higher [24]. The results from this study indicates that students and personnel of this elementary public school are likely exposed to diverse environmental asthma triggers from biological and anthropogenic sources. Anthropogenic sources include combustion products like carbon monoxide (CO), sulfur dioxide (SO_2_) and nitrogen dioxide (NO_2_) from school buses and parents cars in the morning and afternoons and several industrial activities that surround the community. Also VOCs play a significant role in the formation of one of the most damaging pollutants: ground-level ozone. The ozone forms in the presence of sunlight when volatile organic compounds react with nitrogen oxides emitted by cars and industrial plants [22]. At the time of this study there was only one PM_10_ monitoring station at the Municipality. This is a very significant fact because; the Municipality has the highest asthma incidence from zero to 17 year old children. Also, the city have 200,000 habitants, have one of the most crowded traffic highway (PR-3) in the island and three industrial park areas. Reports from the EPA in 2005 mentioned that Carolina was the fourth municipality in the emissions density for 2.5 particulate matter. This situation presents the need of establishing other monitoring station across the Municipality for PM_10_, PM_2.5_ and other air contaminants. Biological triggers include pollen and grass particles since the school is close by several forest areas, and the fact that as a tropical island in the Caribbean temperature and precipitation creates a humid atmosphere with ideal conditions from mold and fungi growth. The school infrastructure and the trends in facilities maintenance are also factors that increase the level of children exposure to asthma triggers. For example, the fact that classrooms do not have air conditioning enhances the possibility of students being exposed to all sorts of triggers from nearby streets and forests. Homes are another setting that children might be exposed to asthma triggers. Our parents survey results indicated that home indoor air quality appear to be clean environment based on answers regarding house cleaning habits, maintenance, and pest presence.

The participating school like almost all public schools in Puerto Rico does not have a nurse assigned or a health and safety assigned personnel. The school does not have a safety committee, maintenance personnel and administrative staff (director and teachers) is not trained in asthma or health safety, and the school director does not have funds or the authority to assign funds for repairs and infrastructure. School maintenance is controlled by the central administration of the department of education in Puerto Rico, and this includes plumbing, electricity, fumigation, and yard maintenance. In addition, many of these services are subcontracts like student transportation that makes impossible to implement environmentally friendly projects like green buses initiatives. This distinct situation of public schools in Puerto Rico calls for initiative that goes beyond the school administration and must involve communities where the schools are located. The results of this work demonstrate the need for such a community based effort to address asthma management.

Successful intervention programs are based on behavioral changes and the social cognitive theory. Some examples of these successful interventions are the one reported by Bayona [25] that address the reduction of tobacco exposure and that reported by Morgan [26] that focused on reducing indoor allergens at homes in urban setting. Another example of a successful community participation initiative in asthma management in a school setting is Awesome Asthma School Days [27]. This Program was created by a community partnership in Milwaukee and uses interactive learning theaters to enhance the educational experience for class attendees. This effort for asthma management included school teachers, family members, medical providers, environmental activists, health plan directors, financial supporters, community leaders, and policymakers. This a health education program for low-income, central city children with asthma. This program improved asthma self-management and school grades for participating children while decreasing the frequency and duration of their asthma episodes. Another successful program is the Neighborhood Asthma Coalition (NAC) [28]. This program was conducted through a well-established neighborhood organization in St. Louis and demonstrated that medical personnel in collaboration with community members could improve awareness of asthma, change attitudes about its care, improve asthma management practices, and reduce the need for acute care for asthma. NAC included educational programs for parents and children, promotional activities, and individualized support provided by trained neighborhood residents. It demonstrated that participation in NAC was associated with positive changes on the Index of Asthma Attitudes scale and lower rates of acute care.

There are other types of initiatives and partnerships that integrate the community’s experiences with the knowledge of the scientific-academic sector. The Michigan Center for the Environment & Children's Health (MCECH) is a community-based participatory research initiative that is also studying environmental, pathophysiological an d clinical mechanisms of childhood asthma and evaluating comprehensive community and household level interventions aimed to reduce asthma-related environmental threats to children, families and neighborhoods. One component of MCECH is the Community Action Against Asthma [29] that coordinates interdisciplinary research that provides benefits to Detroit communities including: identification of previously undiagnosed asthmatic children; provision of household materials, such as vacuum cleaners and clean bedding, aimed at reducing asthma triggers; education on potential asthma triggers and methods for reducing those triggers; assistance to families for negotiation with landlords regarding environmental factors in the home associated with asthma; referrals to families with asthma for available and affordable medical care; the collection of detailed multiple daily measures of ambient and indoor air contaminants; and identification of the underlying mechanisms of asthma and potential targets for further intervention.

Strategies from these interventions can be translated to the school setting. This study provides evidence of the environmental, public health, anthropogenic and socio economic conditions associated with a traditional public elementary urban school in Puerto Rico. For school intervention we suggest the design and implementation of a community based asthma management model that includes the identification of leaders at all levels as well as roles and responsibilities for each component of the community both inside and outside the school. In this work we identified three key elements that must participate and take responsibility in this plan: teachers and administrative staff, students and parents. The model has two main components: communication and call to action. Communication calls for broad dissemination of environmental triggers of asthma and strategies to manage those triggers both in the classroom and at home. This communication must be at all levels from media to local or community newsletters delivered to each home through the school students or by mail. The call to action component requires a strong engagement of the community as a whole meaning that leaders should serve as facilitators instead of doers. Call to action at the school level will require the organization of a school team or committee that will be responsible for the environmental health of the school. Our results showed that school staff is eager to create the committee and 41% of the surveyed parents expressed their willingness to participate as well.

In order to develop a community participation model to address asthma triggers that can be implemented in this school and any school with similar conditions in a tropical setting we integrate all factors related to environmental health and asthma management. The goal of this model is to decrease the children’s asthma episodes by eliminating or avoiding environmental triggers through the active participation of the school community. The proposed community participation model for indoor air quality and asthma management has four phases. First phase consist of the implementation of asthma trigger training workshop for all the potential participating sectors, such as: district superintendents, school administrators, teachers, students and the general community. This must be accomplished in coordination with any environmental health related agencies including the Environmental Protection Agency (EPA), Puerto Rico Lung Association (PRLA), Puerto Rico Department of Health (PRDH), Puerto Rico Department of Education (PRDE), and the Environmental Quality Board (EQB). The second phase consists of organizing a school steering committee that integrates the administration personnel, employees, teachers, parents and students. In this phase, there will be campaign planning, objectives delimitation, development of a work plan, and the role and responsibilities assignment. An ongoing task in this phase is the continuous monitoring and identification of the environmental asthma triggers in the classroom, school, home and community. The third phase is the development of a community specific intervention – call to action plan to reduce exposure to the identified environmental triggers. This plan will include the following activities:

(1) Open house and the celebration of *Days of Clean Air*;
(2) Hosting a health fair at the school for the whole community to participate;
(3) Survey distribution and collection to all the school students and parents to update a precise asthma school profile;
(4) Coordinate in home visits disseminate information in the form of newsletter or bulletin about asthma, indoor air pollutants that act as environmental triggers and alternatives to decrease the exposition to these triggers both at home and outside;
(5) Invite other members of the community to participate in the steering committee and create a train the trainers program so they can involve new members by giving them the opportunity to train other interest citizens, delegate interview opportunities, develop home environmental checklist, participate in air monitoring and become checklist administrators (for their homes, immediate neighborhood or other community surroundings), and develop environmental verification for asthma triggers in their community (identifying potential trigger sources);
(6) Organize a partnership between the EQB and the community to monitor the indoor and outdoor air quality (specially for mold, dust mites, pollen and PM pollution) in the houses, school and in adjacent exterior areas;
(7) Visits to potential environmental trigger sources and share with them information about environmental asthma triggers and suggestions on how this triggers could be minimize or eliminated;
(8) Develop strategies between school administrators, teachers, parents, community leaders, government officials, physicians, and health providers to implement actions that secure asthma free classrooms including hiring a school nurse, creating a law that allow children’s self-medication in the schools or developing a personal plan for asthmatic students to receive their medication in the schools. The fourth and final phase of the recommended model includes the development and implementation of a customized evaluation and assessment plan. This final phase verifies that the objectives have been accomplished, and identify aspects that can be improved considering all participating sectors.

## Conclusions

In this study, we address the potential relationship between anthropogenic and naturally occurring environmental factors, and asthma prevalence in an elementary public school in Carolina, Puerto Rico in an effort to empower and engage communities to work on their environmental health issues.

Climate conditions together with the fact that the largest forest in Puerto Rico is located nearby Carolina suggest that the region has the ideal conditions to promote mold and fungi growth. This natural environment and the anthropogenic activities in the municipality combine to contribute to a diminished air quality. These two increased airborne particulate matter and high levels of VOC both significant sources of asthma triggers. Another important factor in this region is wind, which predominant pattern for Puerto Rico is eastward. Wind patterns can enhance the concentration of air particular matter in the area with contributions from the Sahara dust as well as volcanic ashes from active craters in nearby island like Guadalupe and Montserrat. The geomorphology of the area, the wind patterns and natural resources together with the temperature and precipitation cycles in the zone creates the ideal environmental conditions for increased humidity and pollen, mold and fungi development through the year. Thus, the region is located where natural resources play a significant role in air quality by contributing with biological asthma trigger s. Anthropogenic activities in the region may exacerbate these natural conditions contributing to air pollution and hence the reported high prevalence of asthma in the area.

Our results indicated that male students are more affected by this condition than female students. This contrast significantly with the results obtained from parents and school administrative personnel that showed that females are more affected by asthma than males. This gender and age difference should be further examined in terms of level of outdoor activity, genetic basis and treatment response. The results of this study are higher than those reported by the CDC for Puerto Rico. The latter concurs with previous reports in that higher asthma prevalence is observed in low income, low education background communities, hence underrepresented minority groups. The IAQTFS intervention used in this study showed that this strategy provides a valuable tool to the school community to manage asthma, and could be a potential strategy to use with these groups.

This work validates the fact that Puerto Rico continues to have a significant number of people with asthma, particularly children with asthma. It also demonstrates that schools are important settings to create community based action plans to manage environmental asthma triggers through scientific research, outreach and training. It is recommended to conduct future research on indoor an outdoor air quality monitoring particulary for fungi, dust mites, pollen, PM, NO_2_, and ozone as well as to install other EPA monitoring station in the schools vicinity.
